# Effects of Essential Oils and Some Constituents from Ingredients of Anti-Cellulite Herbal Compress on 3T3-L1 Adipocytes and Rat Aortae

**DOI:** 10.3390/ph14030253

**Published:** 2021-03-11

**Authors:** Ngamrayu Ngamdokmai, Tamkeen Urooj Paracha, Neti Waranuch, Krongkarn Chootip, Wudtichai Wisuitiprot, Nungruthai Suphrom, Kamonlak Insumrong, Kornkanok Ingkaninan

**Affiliations:** 1 Centre of Excellence in Cannabis Research, Department of Pharmaceutical Chemistry and Pharmacognosy, Faculty of Pharmaceutical Sciences and Center of Excellence for Innovation in Chemistry, Naresuan University, Phitsanulok 65000, Thailand; ngamrayun59@nu.ac.th; 2 Department of Pharmacology, Faculty of Pharmacy, Hamdard University, Main Campus, Karachi 74600, Pakistan; tamkeen.urooj@hamdard.edu; 3 Cosmetics and Natural Products Research Center and Center of Excellence for Innovation in Chemistry, Department of Pharmaceutical Technology, Faculty of Pharmaceutical Sciences, Naresuan University, Phitsanulok 65000, Thailand; netiw@nu.ac.th; 4 Department of Physiology, Faculty of Medical Sciences, Naresuan University, Phitsanulok 65000, Thailand; krongkarnc@nu.ac.th; 5 Department of Thai Traditional Medicine, Sirindhorn College of Public Health, Phitsanulok 65130, Thailand; wudtichai@scphpl.ac.th; 6 Department of Chemistry, Faculty of Science and Center of Excellence for Innovation in Chemistry, Naresuan University, Phitsanulok 65000, Thailand; nungruthais@nu.ac.th (N.S.); kamonlaki59@nu.ac.th (K.I.)

**Keywords:** cellulite, essential oil, monoterpenes, Thai herbal compress, adipogenesis, lipolysis, vasorelaxation

## Abstract

Cellulite is associated with a complex array of adipocytes under the skin and vascular system. A herbal compress that was previously developed was proven to have an anti-cellulite effect in healthy volunteers within 2 weeks of treatment. However, its mechanism and ingredients responsible for reducing cellulite were not known. The purpose of this study was to investigate the activity of eight essential oils in, and two water extracts from, the ingredients of the herbal compress together with nine monoterpenoid constituents on the 3T3-L1 adipocytes. The vasodilatory effect on rat aortae was also studied. The adipocytes were induced by dexamethasone, 3-isobutyl-1-methylxanthine and insulin. At all concentrations tested, all essential oils, water extracts and their monoterpenoid constituents significantly inhibited lipid accumulation activity (*p* < 0.05) and decreased the amount of triglycerides when compared to untreated cells (*p* < 0.01). In addition, our results showed that the mixed oil distilled from the herbal compress mixed ingredients could relax the isolated rat aorta (EC_50_ = 14.74 ± 2.65 µg/mL). In conclusion, all essential oils, extracts and chemical constituents tested showed effects on adipogenesis inhibition and lipolysis induction on the cultured adipocytes with the mixed oil demonstrating vasorelaxation activity, all of which might be the mechanisms of the anti-cellulite effects of the herbal compress.

## 1. Introduction

Cellulite is associated with excessive fat accumulation and increases in size and number of adipocytes under the skin, which is caused by genetic, dietary, behavior and hormones. Cellulite is usually found around the thighs and buttocks of post-pubescent females. The increase in these cellulite deposits causes them to invade the dermis which disrupts the tissue architecture, microcirculation, skin elasticity and dermal thickness, resulting in the orange peel-like appearance of the skin [1,2].

There are basically two pathways which can be targeted to achieve cellulite reduction. First, the inhibition of lipogenesis to prevent fat storage in the adipocytes, and, second, the induction of lipolysis which is the metabolic pathway through which lipid triglycerides are hydrolyzed into a glycerol and three fatty acids. Essentially, this is the process of the decomposition of the chemical that causes fat to be released from the adipose tissue by the hydrolysis of the ester bonds in the triglycerides of the fatty tissue under the skin. In addition, the enlarged fat cells, evident as cellulite, lead to the alteration in the microvascular network of the fat tissue, resulting in water retention, which results in the compression of the vascular vessels and in cellular changes [3,4]. There are known compounds, such as retinol, that improve the appearance of cellulite by increasing the microcirculation [5]. The mixture of retinol, caffeine and ruscogenin could increase microcirculation on the thigh of 46 women who showed moderate degrees of cellulite [6].

The use of herbal compresses is popular in traditional Thai therapies, such as in traditional message and spa. These compresses contain herbs bundled within a cloth to form a ball which is warmed and applied to relieve muscle pains, stress and strains. In previous studies [7,8], we modified a Thai traditional herbal compress to use as an anti-cellulite product. The formulation contained *Zingiber officinale* Roscoe rhizomes (ginger), *Piper nigrum* L. fruit (black pepper), *Piper retrofractum* Vahl. Fruit (java long pepper), *Camellia sinensis* (L.) Kuntze leaf (tea) and *Coffea arabica* L. seed (coffee) as the principal ingredients together with some auxiliary herbs i.e., *Zingiber montanum* (J. Koenig) Link ex A.Dietr. rhizomes (Cassumunar ginger or plai), *Curcuma longa* L. rhizomes (turmeric)*, Cymbopogon citratus* DC. Stapf. leaves (lemon grass), *Citrus hystrix* DC. fruit peels (kaffir lime), with camphor and salts added for both scent and skin penetration. In those prior studies, the anti-cellulite effects of the herbal compress were determined via a double-blinded, randomized placebo-controlled trial conducted on 21 female volunteers aged 20 to 55 over an 8-week test period. The results showed that the herbal compress could significantly reduce thigh circumference, skin fold thickness and the severity of cellulite within 2 weeks. However, the mechanisms of this action and the bioactive constituents responsible for such an action were not identified.

In our current study, we distilled the essential oils of the herbal compress and each ingredient, with the tea leaves and coffee beans being extracted by water. All samples were tested, together with their major monoterpenoid constituents (camphor, camphene, citral, 3-carene, limonene, myrcene, alpha-pinene, beta-pinene and terpinene-4-ol), for their anti-cellulite effects. The effects of these samples on lipid accumulation were demonstrated, in vitro, on the mouse adipocyte cell 3T3L1 model and the inhibiting of adipogenesis and stimulation of lipolysis was observed and measured. Further, the vasorelaxant effect of the mixture of the essential oil, distilled from the powdered form of all ingredients, was tested on the aortae isolated from rats.

## 2. Results and Discussion

### 2.1. Cell Viability

Notably, 3T3-L1 adipocytes are widely used in assays of adipogenesis because they can tolerate an increased number of passages and homogeneously respond to treatments [9,10]. Prior to our study of the effects of essential oils/extracts and their major monoterpenoid constituents on adipogenesis and lipolysis of 3T3-L1 adipocytes, we tested the viability of the various concentrations (1–500 μg/mL) of the samples on the preadipocytes and adipocytes.

In addition, the viability of the samples on keratinocyte, fibroblast was studied to provide safety information. The highest dilution that resulted in more than 80% cells being viable was considered to be the non-toxic concentration to be used in our further studies on adipogenesis and lipolysis of 3T3-L1 adipocytes. Table 1 illustrates the non-toxic levels of the tested concentrations.

### 2.2. Preventive and Treatment Effects of Essential Oils/Extracts and Their Major Monoterpenoid Constituents on Adipogenesis of 3T3-L1 Cells

Adipogenesis is a complex process by which pre-adipocytes transform into adipocytes. Oil red O staining is the most commonly used method for distinguishing adipocytes from other cells and has recently been used as a quantitative method to assess different degrees of adipocyte differentiation [11]. In our study, the pre-adipocytes were treated with dexamethasone, 3-isobutyl-1-methyl xanthine (IBMX) and insulin to induce the differentiation. After nine days, the formation of adipocytes was evaluated. The preventive and treatment effects of the essential oils distilled from the ingredients of the herbal compress and the aqueous extracts of tea and coffee on adipogenesis were studied at the concentrations that were non-toxic to the cells. To investigate the preventive effect, the samples were added to the media on day 3, 5 and 7 after the initiation. Their effects on 3T3-L1 adipocyte differentiation were observed via lipid accumulation oil red O staining on day 9. To evaluate the treatment effect, the samples were incubated with the mature adipocyte on day 9 after the initiation and their effects were measured on day 10.

**Figure 1:**
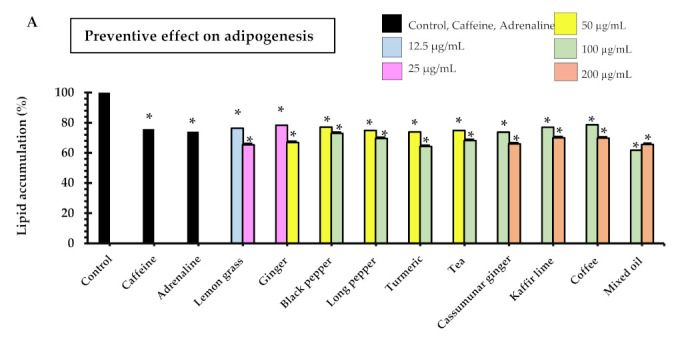

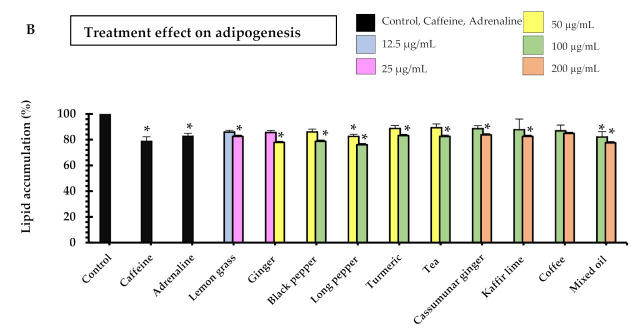
Lipid accumulation in 3T3-L1 adipocytes after treated with essential oil, extracts and positive controls (adrenaline 0.1 mM or 18.3 µg/mL and caffeine 1 mM or 194.2 µg/mL); (**A**) in the preventive experiments where the pre-adipocytes were treated with the samples during the differentiation on days 3, 5 and 7, and (**B**) in the treatment experiments where the samples were added after the pre-adipocytes were differentiated to adipocytes (on day 9) and incubated for one day. The lipid accumulation was measured by Oil Red O assay, and the results are expressed as the mean ± SEM of triplicate tests. Data expressed in percentage in comparison with control. One-way ANOVA showed significant value, * *p* < 0.05 as compared to control.

The results showed that all samples had preventive effects on adipogenesis in a dose-dependent manner. Of the samples tested, lemon grass oil demonstrated inhibition of lipid accumulations at a concentration of 12.5 µg/mL that was 23 ± 6%, which was the lowest effective concentration of all samples tested (Figure 1A). As well as lemon grass, another promising sample was ginger oil, which gave 33 ± 5% inhibition at the concentration of 50 µg/mL. The remaining essential oils and extracts tested showed around 30% inhibition at the concentration of 100–200 µg/mL, whereas the positive controls i.e., 18 µg/mL adrenaline and 194 µg/mL caffeine expressed 24 ± 5% and 25 ± 2% inhibition, respectively. Manaharan et al. 2016 reported that ginger oil at various concentrations (50 to 800 µg/mL) significantly decreased lipid content in mature adipocytes in a dose-dependent fashion [12]. Coffee and tea have shown anti-obesity and anti-adiposity activities in adipocytes in some previous studies [13,14]. Both coffee extracts and their major constituents, namely caffeine, caffeic acid, chlorogenic acid and trigonelline, increased glycerol release while also reducing the accumulation in the 3T3-L1 cells during adipocytic differentiation. Also, the expression of the peroxisome proliferator-activated receptor γ (PPARγ), a transcription factor that controls the differentiation of adipocytes, is inhibited by the consumption of coffee. Also, as reported by [15,16], the main theaflavin of tea, polyphenols and theaflavin-3,3′-digallate (TF3), demonstrated an anti-adiposity effect in mature adipocytes through the activation of the AMPK pathway. Further, Goto et al. [17] showed that several bioactive terpenoids, which are derived from herbal and dietary plants, function as PPAR modulators as regulators of carbohydrate and lipid metabolism. However, the anti-adipogenic effects of the essential oils distilled from lemongrass, black pepper, long pepper, turmeric and cassumunar ginger, as well as the mixed oil from herbal compresses, are reported here for the first time.

The lipid accumulation, after the mature adipocytes had been treated with samples of the essential oils and tea and coffee extract, was evaluated to ascertain the effect of the treatment. Figure 1B shows that lemon grass (25 µg/mL), ginger (50 µg/mL), black pepper (100 µg/mL), long pepper (50, 100 µg/mL) and mixed oil (100, 200 µg/mL) significantly inhibited lipid accumulation in the range of 12–24%. Interestingly, the positive controls i.e., 18 µg/mL adrenaline and 194 µg/mL caffeine showed the same range of % lipid accumulation inhibition (21% and 17%, respectively). All samples tended to decrease intracellular lipids in a concentration-dependent manner. The maximum inhibition of lipid accumulation (24%) was observed from 100 µg/mL long pepper oil. It is noted that the positive controls as well as all test samples could reduce lipid accumulations on 3T3-L1 adipocytes in both preventive and treatment experiments where the degree of reduction was greater in the preventive experiments. The samples that clearly showed significantly higher % lipid accumulation in the preventive experiments when compared to the treatment experiments were turmeric, cassumunar ginger, tea and mixed oil (*p* < 0.05).

**Figure 2:**
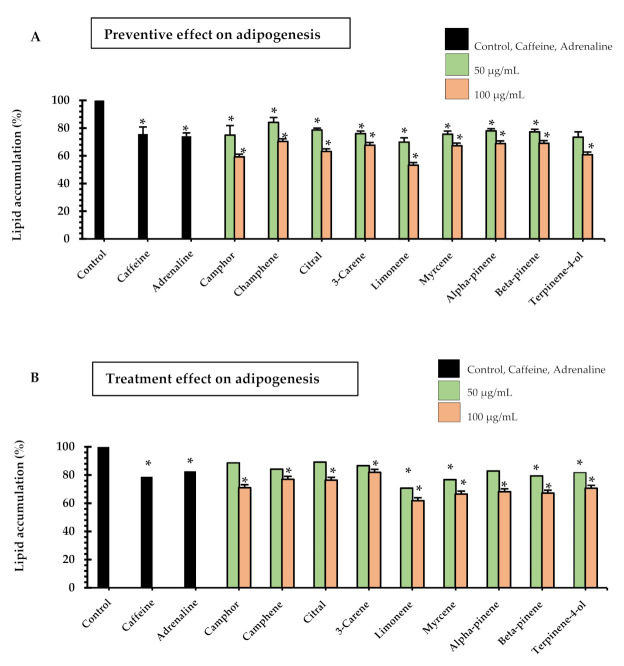
Lipid accumulation in 3T3-L1 adipocytes after treated with monoterpenoid constituents of the herbal compress ingredients and positive controls (adrenaline 0.1 mM or 18.3 µg/mL and caffeine 1 mM or 194.2 µg/mL); (**A**) in the preventive experiments where the pre-adipocytes were treated with the samples during the differentiation on days 3, 5 and 7, and (**B**) in the treatment experiments where the samples were added after the pre-adipocytes were differentiated to adipocytes (on day 9) and incubated for one day. The lipid accumulation was measured by Oil Red O assay, and the results expressed as the mean ± SEM of triplicate tests. Data expressed in percentage in comparison with control. One-way ANOVA showed significant value, * *p* < 0.05 as compared to control.

**Figure 3:**
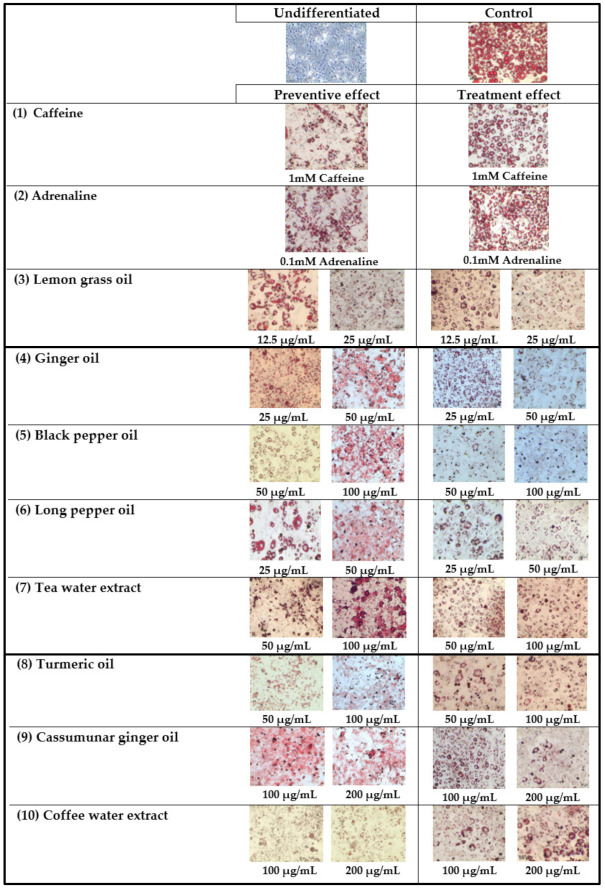

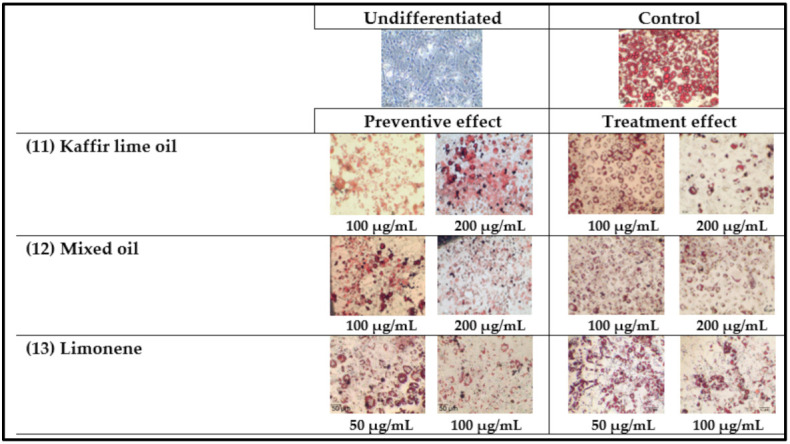
Representative photographs (20×) of 3T3-L1 adipocytes stained with Oil Red O after treatment with the essential oils, extracts and limonene in comparison with undifferentiated and control cells. To study the preventive effect, the mature adipocytes were treated with samples (1–13) on days 3, 5 and 7 after the initiation and the oil red O staining was conducted on day 9. For treatment effect, the samples were incubated with the mature adipocyte on day 9 and their effects were measured on day 10. Notably, 1 mM (18.3 µg/mL) caffeine and 0.1 mM (194.2 µg/mL) adrenaline were used as positive controls.

Nine monoterpenoid constituents of the herbal compress ingredients were tested for their preventive and treatment effects on adipogenesis of 3T3-L1 adipocytes (Figure 2). The results showed that all samples significantly inhibited lipid accumulation as compared to the control cells in both preventive and treatment ways, although most samples tended to have a higher preventive effect than the treatmentive effect. The significant difference between preventive effects and treatment effects are shown in citral (50, and 100 µg/mL, *p* < 0.001), and 3-carene (100 µg/mL, *p* < 0.05). For the effective effect, the highest inhibition of lipid accumulation was observed in limonene at the concentration of 100 µg/mL, with 47 ± 2% inhibition. The limonene compound significantly decreased lipid accumulation more than the caffeine (194.2 µg/mL, 38 ± 1% inhibition) and adrenaline (18.3 µg/mL, 34 ± 3% inhibition). For the treatment effect of the intracellular lipid accumulation, these results indicated that the nine major monoterpenoid constituents inhibited lipid accumulation (Figure 2B). These data also show that camphor, camphene, citral, 3-carene, alpha-pinene in the concentration of 100 µg/mL as well as limonene, myrcene, beta-pinene and terpinene-4-ol in the concentrations of 50 and 100 µg/mL significantly inhibited lipid accumulation by 20–33% where caffeine (18 µg/mL) showed 18 ± 1% inhibition and adrenaline (194 µg/mL) showed 21 ± 3% inhibition. The highest % inhibition of lipid accumulation was observed in limonene at 100 µg/mL (38 ± 4%), which was significantly higher than caffeine and adrenaline (*p* < 0.05). A previous animal study [18] showed the preventive effects of limonene on hyperglycemia and dyslipidemia in high-fat diet-induced obesity mice. In addition, limonene was reported to be effective in regulating the peroxisome proliferator-activated receptor (PPAR)-α signaling and liver X receptor (LXR)-β signaling. The microscopic pictures of 3T3-L1 adipocytes after stained with Oil Red O in preventive and treatment experiments are shown in Figure 3.

### 2.3. Effects of Essential Oils/Extracts and Their Major Monoterpenoid Constituents on Triglyceride Accumulation of Adipogenesis of 3T3-L1 Cells

**Figure 4:**
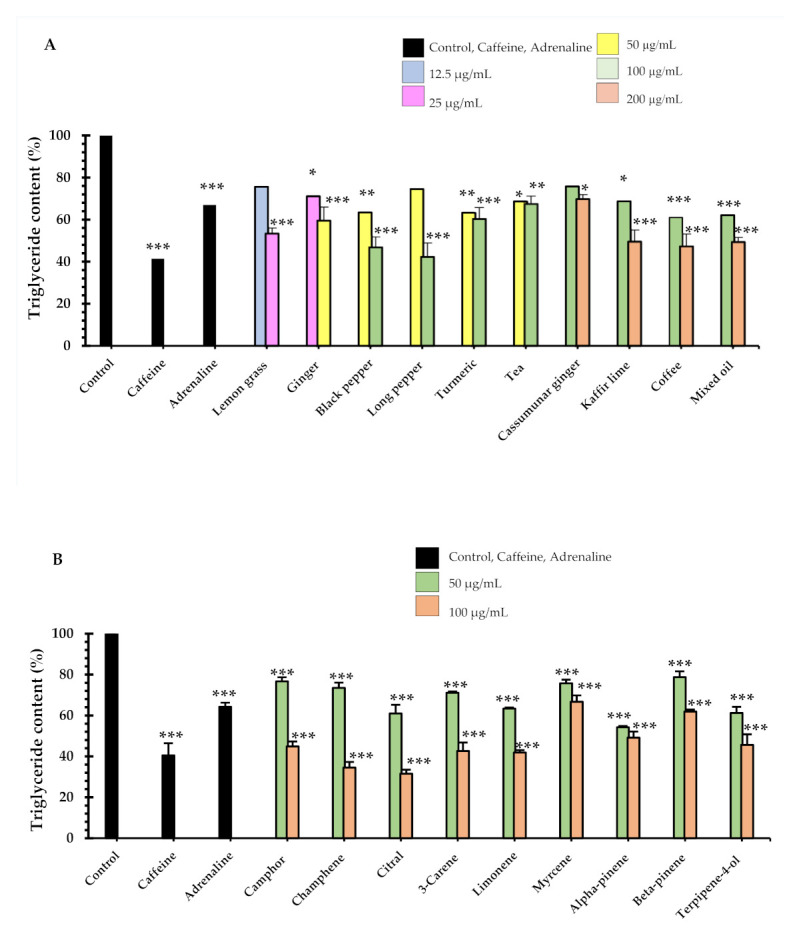
The effects of (**A**) the essential oils/extracts, and (**B**) monoterpenoid constituents on triglyceride content of 3T3-L1 adipocytes after treatment for 24 h. Intracellular triacylglycerol content was determined using enzymatic colorimetric methods. We used 1 mM (18.2 µg/mL) caffeine and 0.1 mM (194.3 µg/mL) as positive controls. Values are expressed as mean ± SE of three independent experiments. One-way ANOVA showed significant value, * *p* < 0.05, ** *p* < 0.01, and *** *p* < 0.001 vs. control cells (untreated).

In addition to inhibition of adipocyte differentiation and mature adipocyte, we also determined the effect of the test samples on triglyceride accumulation of 3T3-L1 adipocytes causing the in vitro lipolysis effect. Excessive amounts of triglyceride accumulation in the adipocyte is related to an increased risk of a variety metabolic disease. In our study, treatment of cells with seven essential oils, mixed oil and tea and coffee extracts decreased triglyceride accumulation in differentiated 3T3-L1 cells. The amount of intracellular triglyceride accumulated in adipocytes was significantly decreased at all concentration levels tested (Figure 4A,B) when compared to the effect in the untreated control cells.

The lowest concentration that significantly decreased triglyceride content was observed in lemon grass oil (25 µg/mL, 53 ± 3% triglyceride content). Five essential oils that demonstrated the most prominent decrease for the triglyceride content were long pepper oil (100 µg/mL, 42 ± 6% triglyceride content), black pepper (100 µg/mL, 47 ± 5% triglyceride content), coffee (200 µg/mL, 47 ± 7% triglyceride content), kaffir lime (200 µg/mL, 50 ± 5% triglyceride content) and mixed oil (200 µg/mL, 50 ± 2% triglyceride content). A total of 1 mM of Caffeine decreased the triglyceride contents by 41 ± 2% and 0.1 mM adrenaline decreased triglyceride accumulation by 67 ± 4% (relative to the control).

For nine monoterpenoid constituents on lipolysis of lipid accumulation in adipocytes, at day 10, were measured using intracellular triglyceride content, as shown in Figure 4B. The maximum significant decrease of triglyceride content was identified in the presence of citral (100 µg/mL concentration, 32 ± 1% triglyceride content) and camphene (100 µg/mL, 35 ± 2% triglyceride content), relative to the control. In addition, two constituents: camphene and citral at 100 µg/mL concentrations, decreased triglyceride content more than caffeine (*p* < 0.01). We also found that nine constituents had decreased triglyceride content more than adrenaline. These were camphor, camphene, citral, 3-carene, limonene, myrcene, alpha-pinene, beta-pinene and terpinene-4-ol at 100 µg/mL concentration. Interestingly, triglyceride determination of camphene, citral and limonene showed similar decreases in total lipid accumulation in mature adipocytes as compared to the 1% dimethyl sulfoxide (DMSO) control. Our results concur with the animal study reported in [19], which showed that there was a significant inhibition of differentiation of preadipocytes to mature adipocytes was observed, and it was evident from reduced lipid accumulation in the cells. Cellular lipid content was decreased by 18% by camphene at 10 µM, by 29% at 50 µM and by 37% at 100 µM, when compared with the control cells treated with DMSO. Previous work has also found that 30 µM citral exhibits significant inhibition of total triglyceride accumulation using the triglyceride determination kit by 30%, while 40 µM showed a 50% inhibition, and 50 µM showed 80% inhibition [20]. In another study [21], a quick screening on the lipolytic effect of monoterpenes in 3T3-L1 adipocyte was conducted with the result that 1µM of limonene stimulated lipolysis by 17%. Caffeine and adrenaline were used as a positive control in this study. Figure 4A,B show that caffeine at the concentration of 1 mM or 194 µg/mL increased lipolysis with a triglyceride accumulation reduction of 41 ± 2% triglyceride content. Adrenaline at the concentration of 0.1 mM or 18 µg/mL also decreased triglyceride accumulation (67 ± 4% triglyceride content). These results are supported by a previous report that showed that caffeine inhibited triglyceride content by 11%, 22% and 34% at the concentration of 1, 5 and 10 µg/mL [22]. In addition, adrenaline (1 µM) stimulated lipolysis for about a 30% glycerol release. Total glycerol content in the medium indicates the lipolytic effect of adrenaline in 3T3-L1 adipocytes [21]. Previous studies demonstrated that lemon grass oils are rich in citral [23,24], and also that citral inhibited the formation of intracellular lipid accumulation in a concentration-dependent manner (10–50 µM) for 30, 40 and 50 µM concentrations of citral [20]. It should be noted that the present research has shown the potent impact on the lipolytic effect of nine essential oils, mixed oil and their major constituents, which are monoterpenes, The potential anti-cellulite activity of the seven essential oils, mixed oil and tea and coffee water extracts, and their nine major monoterpenoid constituents, extracted from the anti-cellulite herbal compress that we had developed, were assayed on the 3T3-L1 cell lines of preadipocyte, a commonly used cell model for adipose cell biology research. Furthermore, anti-cellulite effects can be exerted by reducing the size and number of intracellular lipid accumulations in adipocytes and assayed triglyceride accumulations. It is suggested that further work be undertaken to study the molecular mechanisms and to substantiate the effectiveness of bioactive compounds as anti-lipogenesis or lipolysis substances, and the beneficial anti-cellulite activities that we have shown to be demonstrated by the herbal compress.

### 2.4. Vasorelaxant Effects Of Mixed Oil on Rat Aortae

**Figure 5:**
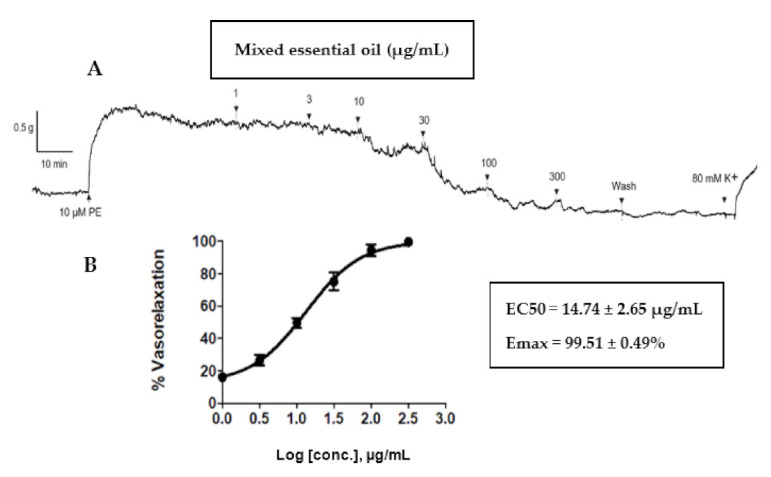
(**A**) A typical trace of the vasorelaxant effect of the mixed oil distilled from the herbal compress mixed ingredients (0–300 μg/mL) on endothelium-intact aortae. (**B**) Concentration-relaxation curves for mixed oil from anti-cellulite herbal compress (1–300 μg/mL) on endothelium-intact aortic rings. All data points are means ± SEMs (*n* = 5).

**Figure 6:**
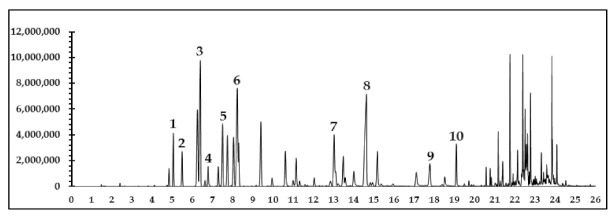
GC-MS total ion chromatogram of the mixed oil distilled from the herbal compress mixed ingredients. The peaks of the main constituents were identified by comparing the molecular mass from mass spectra data of each compound with the NIST library (Version 2.2) as (1) α-pinene, (2) camphene, (3) β-pinene, (4) β-myrcene, (5) 3-carene (6) D-limonene, (7) camphor, (8) terpinene-4-ol, (9) β-citral and (10) α-citral.

Our previous clinical study showed that the anti-cellulite herbal compress improved cellulite appearance, as assessed by measurement of thigh circumferences, skin thickness and severity of cellulite [7]. Blood flow enhancement via vasodilation could be one of the mechanisms activated by the anti-cellulite herbal compress. Therefore, the mixed oil derived from hydrodistillation of the herbal compress was tested on isolated rat aortae. This test revealed a concentration-dependent (1–300 μg/mL) vasorelaxant effect of mixed oil on the phenylephrine pre-contracted endothelium-intact vessel (EC_50_ = 14.74 ± 2.65 µg/mL and E_max_ = 99.51 ± 0.49%, Figure 5). The vascular action of the herbal compress could be attributed to some of the major constituents of mixed oil, in particular monoterpenes i.e., α-pinene, camphene, β-pinene, β-myrcene, 3-carene, D-limonene, camphor, terpinene-4-ol β-citral and α-citral, which were displayed on chromatographic profiles shown in a GC-MS chromatogram (Figure 6). The mechanism of vasorelaxant actions of the mixed oil could involve the endothelium dependent pathway i.e., nitric oxide release, as reported by several studies evaluating vascular actions of the volatile oils and plants containing similar monoterpene profiles [7,25,26].

## 3. Materials and Methods

### 3.1. Chemicals and Plant Materials

Keratinocyte serum-free medium (KSFM) and supplements (2.5 μg of recombinant human epidermal growth factor and 25 mg of bovine pituitary extract), high glucose Dulbecco’s Modified Eagle’s Medium (DMEM), bovine calf serum (BCS), fetal bovine serum (FBS), phosphate buffered saline (PBS) and antibiotics were purchased from GIBCO (Grand Island, NY, USA), while 3-(4,5-dimethylthiazol-2-yl)-2,5-diphenyl-tetrazolium bromide (MTT), DMSO, Oil Red O reagent and human recombinant insulin were purchased from Sigma-Aldrich (St. Louis, MO, USA. Dexamethasone and IBMX were purchased from Merck (Kenilworth, NJ, USA). Caffeine, camphene, camphor, 3-carene, α-citral, β-citral, limonene, β-myrcene, α-pinene, β-pinene and terpinene-4-ol were purchased from Sigma-Aldrich (Buchs, Switzerland). Adrenaline was purchased from MARCH (Bangkok, Thailand).

The ingredients of the herbal compress i.e., ginger (rhizome), black pepper (fruit), java long pepper (fruit), turmeric (rhizome), plai (rhizome), lemongrass (stalk) and kaffir lime (fruit peel) were purchased in Phitsanulok, Thailand. Specimens of all nine herbs were collected and authenticated by comparing with voucher lots available in the Biological Sciences Herbarium, Naresuan University, Phitsanulok or comparing with botanical illustrations. Roasted coffee beans (Arabica100% Coffman^®^) were produced by Coffman International. Co., Ltd., Bangkok, Thailand and the tea (Three Horses^®^) was purchased from Three Horses Tea Co., Ltd., Bangkok, Thailand.

### 3.2. Extraction

Essential oil extraction: The essential oil of each seven plant ingredients as well as the mixed oil were extracted using hydro-distillation. The plant materials were cut into small pieces, dried at 45–50 °C and ground into powder. The powder of each plant (50 g) was placed in a round-bottomed flask with 500 mL of distilled water. For the mixed oil, the 150 g of mixture of seven plant herbal compress ingredients in the ratio that reported in previous study [7] was placed in a round-bottomed flask with 1500 mL of distilled water. The distillation apparatus was set to 100 °C for 3 to 5 h of distillation [27,28]. Tea and coffee water extraction: Tea and coffee (100 g) were boiled in 400 mL of distilled water for 3 times, and filtered through a filter cloth, followed by 5 min of centrifuging, then the supernatant was lyophilized and stored at −20 °C in screw cap bottles.

### 3.3. Cell Culture

The protocol was approved by Naresuan University Institutional Review Board for human keratinocyte and fibroblast cells (Approval number 608/59), and for 3T3-L1 preadipocytes and adipocytes (Approval number 0044/61). 3T3-L1 preadipocyte cell line was obtained from ATCC (Manassas, VA, USA). Keratinocyte cells were cultured in KFSM supplemented with 5 µg/mL epidermal growth factor human recombinant, 50 μg/mL bovine pituitary extract and 1% P/S solution [29]. Fibroblast and 3T3-L1 preadipocyte cells were cultured in DMEM, 10% FBS (fibroblast), or 10% BCS (3T3-L1), 3.7 g/L sodium bicarbonate and 1% P/S solution. The cells culture condition was maintained at 37 °C, and humidified in an atmosphere of 5% CO_2_.

### 3.4. Adipocyte Differentiation (Adipogenesis Assay)

For adipocyte differentiation, the 3T3-L1 pre-adipocytes were plated in 96 well plates at a density of 2 × 10^3^/well and cultured in DMEM supplemented with 10% BCS and 1% P/S solution. Two days after confluence; day 0, the media was removed and the fresh differentiation media i.e., DMEM with 10% FBS, 1 μM dexamethasone, 0.5 mM IBMX and 5 μg/mL insulin was added and maintained for 2 days at 37 °C in an atmosphere of 5% CO_2_. The media was replaced with fresh DMEM containing insulin every second day. By day 9, more than 90% of the cells had differentiated into lipid droplets [30,31].

### 3.5. Cell Viability

Human keratinocyte and fibroblast cells were separated from human foreskins. The keratinocytes were seeded at 2 × 10^4^ cells/well and the fibroblast cells were seeded at 2 × 10^4^ cells/well. The 3T3-L1 pre-adipocytes were seeded at 1 × 10^4^ cells/well and 3T3-L1 pre-adipocytes, for differentiation to the adipocytes, were seeded at 2 × 10^3^ cells/well in a 96-well plate. The essential oils and extracts and their major compounds were dissolved in 100% DMSO and the culture medium was then replaced with 100 µL serial dilutions (0.97, 1.95, 3.90, 7.81, 15.62, 31.25, 62.5, 125, 250 and 500 µg/mL) of the extracts for keratinocytes, fibroblast and pre-adipocytes, and 12.5, 25, 50, 100, 200 and 500 µg/mL for the adipocytes. The cells were then incubated with the essential oils/extracts at 37 °C, and 5% CO_2_, for 24 h. The viability of the differentiated cells adipocyte and post-confluent adipocytes was ascertained by treating them with a sample solution in differentiation medium every 2 days for 9 days, after which the viability was assayed. Where the final concentration of DMSO was less than 1% *v*/*v* in the cell culture medium, the cells were added to each well with 50 µL of MTT working solution (1 mg/mL) in PBS (pH = 7.4) and incubated for a further three hours. The solution was measured at a test wavelength of 595 nm by microplate reader [32,33,34].

### 3.6. Quantification of Lipid Content by Lipid Accumulation

The accumulation of lipids in the cells was quantified by Oil-Red-O assay. The inhibition of essential oils and extracts, and their major monoterpenoids, were evaluated for their preventive and treatment effects against adipogenesis. Various concentrations of the essential oils and extracts (12.5 to 200 μg/mL) were added to the differentiation media (with insulin) on days 3, 5 and 7. After day 7, the lipid droplets in the mature adipocytes were stained then visualized through Oil Red O staining and photographed. To evaluate the treatment effect, the samples were incubated with the mature adipocyte on day 9 for 24 h and their effects were stained with Oil Red O on day 10 [12,20].

### 3.7. Oil-Red-O Staining

After the lipid droplets were stained and showed through the Oil Red O staining of 3T3-L1, the adipocytes were treated with different concentrations of the extracts, as described above. The cells were washed twice with PBS, fixed with 10% formalin for 8 min and left for 1 h at room temperature when they were washed again with 60% Isopropanol and stained with freshly prepared Oil Red O solution diluted with 3 parts of 0.5% Oil Red O in 2 parts of distilled water, for 45 min at room temperature. The cells were again washed twice with distilled water to remove the excess stain and then were dried. The cells were then examined under a microscope. After 10 min, the Oil Red O staining was extracted by isopropanol. The absorbance was measured using a microplate reader at 500 nm [31,35] and examined under a microscope (Nikon) and the images were captured.

### 3.8. Determination of Triglyceride (TG) Content

Next, 3T3-L1 adipocytes were induced for differentiation in the same fashion as stated in session 3.5. At day 9, they were treated with samples of essential oils, extracts and monoterpenoids, or positive controls of caffeine and adrenaline, and incubated at 37 °C, and 5% CO_2_ for 24 h. The cells were then collected and lysed using sonication. The total triglyceride contents in the cells were determined using the Triglyceride Assay Kit (Cayman Chemical Company, Ann Arbor, MI, USA) [20,35,36].

### 3.9. Vasorelaxant Effects of Mixed Oil

This study was approved and conducted in accordance with the guidelines of the Naresuan University Animal Care and Use Committee (NUACUC; Animal Ethics Approval Number: NU-AE601021). After anesthetizing the male Wistar rats, using intraperitoneal injection of thiopental sodium (100 mg/kg), the rats’ aortae were excised and kept in cold physiological Krebs’ solution (mM): NaCl 122 mM; KCl 5 mM; [N-(2-hydroxyethyl) piperazine N’-(2-ethanesulfonic acid)] HEPES 10 mM; KH_2_PO_4_ 0.5 mM; NaH_2_PO_4_ 0.5 mM; MgCl_2_ 1 mM; glucose 11 mM; and CaCl_2_ 1.8 mM (pH = 7.4). After removal of the superficial connective tissues, each aorta was cut into ring segments, 3–4 mm in length, which were then mounted in standard 10 mL organ baths continuously aerated (95% O_2_:5% CO_2_) and filled with Krebs-Hensleit (KH) buffer (pH = 7.4) at 37 °C. A Mac Lab A/D converter (Chart V5, A.D. Instruments, Castle Hill, NSW, Australia) was used to measure the isometric tension of the force transducers which were connected with intra-luminal wires. The resting tension of the aortic rings was maintained at 1 g and the rings were equilibrated for 60 min to ensure a stable contraction with 10 µM phenylephrine (PE). Presence of the endothelial lining was evaluated by observing >70% relaxation with 10 µM acetylcholine (Ach) after stable contraction with PE [37].

The experiment was conducted on endothelium intact rat aortae equilibrated at 1 g initially and pre contracted with 10 µM PE. A stable contraction plateau was observed which was then followed by the cumulative addition of mixed oil from 1 μg/mL, 3 μg/mL, 10 μg/mL, 30 μg/mL, 100 μg/mL and 300 μg/mL. Each concentration was incubated until the relaxation was stable. Identical concentrations of DMSO alone were added to serve as the negative control group at the same time interval as the addition of the mixed oil, in order to ensure that the relaxation was rendered by the mixed oil rather than the DMSO. The vessel was washed with physiological Kreb’s solution after complete relaxation had been observed at the highest concentration of the mixed oil. To evaluate the vessels’ integrity, 80 mM K^+^ solution was added. The immediate contraction plateau of each vessel was observed, signifying the vessel’s viability throughout the experimental protocol. The % relaxation was calculated as % contraction in response to PE.

### 3.10. Gas Chromatography-Mass Spectrometry Analysis of Monoterpenoid Constituents in Mixed Oil

GC-MS analysis used an Agilent 7890B, Gas Chromatography System-5977B coupled to an Agilent 5977B MSD model mass spectrometer (Agilent Technologies, Singapore). Mixed oil was prepared by dissolving 5 mg into 1 mL of methanol and injected into a capillary column HP-5 5% Phenyl Methyl Silox (30 m × 250 μm × 0.25 μm; Agilent 19091S-433) with a constant flow rate of Helium 1.0 mL/min. The injector was set at 250 °C and performed by split mode with a split ratio of 100:1 (in 1.0 μL). The GC oven temperature was initially set at 70 °C for 5 min, then increased to 100 °C at a rate of 3 °C/min and held for 3 min, then increased to 250 °C at a rate of 20 °C/min and held for another 1 min, with a total run time of 26.5 min (Figure 6).

Monoterpenoid constituents of the mixed oil were identified by mass spectrometry in full scan mode using mass analyzer and confirmed by comparing their spectra to those of the NIST MS search 2.2 library. The mass spectrometer was operated in the electron impact ionization mode (70 eV), with a scan range of 50 to 550 amu. The interested constituents of mixed oil from the anti-cellulite herbal compress and their relative peak areas were listed in Table 2.

### 3.11. Statistical Analysis

All adipogenesis experiments, each with a set of 3 wells, were carried out in triplicate. Data were statistically evaluated by a one-way analysis of variance (ANOVA). Determination of significant differences (*p* < 0.05) between means was supported by Tukey’s multiple comparison test (GraphPad Prism software version 8.0, San Diego, CA, USA). Values are given as mean ± standard error of the sample animals. The EC_50_ values and Emax values to achieve maximum relaxation were obtained by concentration-response curve fitting using GraphPad Prism software version 8.0, San Diego, CA, USA.

## 4. Conclusions

This study presents the preclinical effects, on cellular lipid accumulation, triglyceride content and the vasodilatation effect of on rat aortae, of the essential oils and extracts obtained from Thai traditional herbal compresses and their constituents. These findings demonstrate the abilities of the test samples to decrease lipid accumulation resulting in the inhibition of adipocyte differentiation and increasing lipolysis on 3T3-L1 adipocyte cells. The mixed oils showed vasodilatory effects on rat aortae via endothelium-dependent release of vasodilators. Our study is the first to report on the anti-cellulite mechanisms of Thai traditional herbal compresses, including prevention of lipid accumulation and increasing blood flow. Our findings allow us to confidently suggest that the anti-cellulite activity of volatile oils and their monoterpenes constituents, or combinations of them, are useful in the treatment for cellulite.

## Figures and Tables

**Table 1:** Non-toxic concentrations of 7 essential oils, mixed oil, tea and coffee extracts, and their major constituents in anti-cellulite herbal compress selected from 3-(4,5-dimethylthiazol-2-yl)-2,5-diphenyl-tetrazolium bromide (MTT) assay.

Samples	Concentrations (μg/mL)			
	KR ^1^	FB ^2^	PA ^3^	A ^4^
1. Lemon grass oil	31.25	31.25	31.25	62.5
2. Ginger oil	62.5	125	62.5	125
3. Black pepper oil	125	125	125	125
4. Long pepper oil	125	250	125	250
5. Tea water extract	125	250	125	250
6. Turmeric oil	125	250	125	250
7. Cassumunar ginger oil	250	250	250	250
8. Coffee water extract	250	250	250	250
9. Kaffir lime oil	250	250	250	250
10. Mixed oil	250	250	250	250
11. Camphor	ND	ND	200	200
12. Camphene	ND	ND	200	200
13. Citral	ND	ND	200	200
14. 3-carene	ND	ND	200	200
15. D-limonene	ND	ND	200	200
16. β-myrcene	ND	ND	200	200
17. α-pinene	ND	ND	200	200
18. β-pinene	ND	ND	200	200
19. Terpinene-4-ol	ND	ND	200	200
20. Caffeine	1 mM (194.2 µg/mL)			
21. Adrenaline	0.1 mM (18.3 µg/mL)			

^1^ Keratinocyte; ^2^ Fibroblast; ^3^ 3T3-L1 preadipocyte; and ^4^ adipocyte cells. (ND = not determined).

**Table 2:** The GC-MS retention times and the relative peak areas of the interested monoterpenoid constituents of mixed oil from the anti-cellulite herbal compress.

Monoterpenes Constituents ^1^	Retention Time (min)	Relative Area (%) ^2^
α-pinene (1)	5.044	6.13
Camphene (2)	5.478	3.73
β-Pinene (3)	6.383	20.99
β-myrcene (4)	6.774	2.63
3-carene (5)	7.495	8.34
D-limonene (6)	8.221	20.70
Camphor (7)	13.036	8.38
Terpinene-4-ol (8)	14.630	20.93
β-citral (9)	17.791	3.84
α-citral (10)	19.108	4.33

^1^ Relative area (%) obtained by area of the interested peak/total area of 10 interested peaks × 100. ^2^ The number in the brackets of represent the peak in Figure 6.

## Data Availability

The data presented in this study are available on request from the corresponding author.
